# Experimental investigation and analytical verification of buckling of functionally graded carbon nanotube-reinforced sandwich beams

**DOI:** 10.1016/j.heliyon.2024.e28388

**Published:** 2024-04-02

**Authors:** Emrah Madenci, Yasin Onuralp Özkılıç, Alireza Bahrami, Ceyhun Aksoylu, Muhammad Rizal Muhammad Asyraf, Ibrahim Y. Hakeem, Alexey N. Beskopylny, Sergey A. Stel'makh, Evgenii M. Shcherban, Sabry Fayed

**Affiliations:** a Department of Civil Engineering, Necmettin Erbakan University, 42090, Konya, Turkey; b Department of Civil Engineering, Lebanese American University, Byblos, Lebanon; c World Class Research Center, Advanced Digital Technologies, State Marine Technical University, Saint Petersburg, 190121, Russia; d Department of Building Engineering, Energy Systems and Sustainability Science, Faculty of Engineering and Sustainable Development, University of Gävle, 801 76 Gävle, Sweden; e Department of Civil Engineering, Konya Technical University, 42250, Konya, Turkey; f Engineering Design Research Group, Faculty of Mechanical Engineering, Universiti Teknologi Malaysia, 81310, Johor Bahru, Johor, Malaysia; g Department of Civil Engineering, College of Engineering, Najran University, Najran, Saudi Arabia; h Department of Transport Systems, Faculty of Roads and Transport Systems, Don State Technical University, 344003, Rostov-on-Don, Russia; i Department of Unique Buildings and Constructions Engineering, Don State Technical University, Gagarin Sq. 1, 344003, Rostov-on-Don, Russia; j Department of Engineering Geology, Bases, and Foundations, Don State Technical University, 344003, Rostov-on-Don, Russia; k Civil Engineering Department, Faculty of Engineering, Kafrelsheikh University, Kafr El Sheikh, 33516, Egypt

**Keywords:** Composite sandwich beam, Carbon nanotube, Carbon fiber-reinforced polymer, Glass fiber-reinforced polymer, Buckling, Flexural behavior, Strength, Stiffness

## Abstract

Carbon nanotube (CNT) reinforcement can lead to a new way to enhance the properties of composites by transforming the reinforcement phases into nanoscale fillers. In this study, the buckling response of functionally graded CNT-reinforced composite (FG-CNTRC) sandwich beams was investigated experimentally and analytically. The top and bottom plates of the sandwich beams were composed of carbon fiber laminated composite layers and hard core. The hard core was made of a pultruded glass fiber-reinforced polymer (GFRP) profile. The layers of FG-CNTRC surfaces were reinforced with different proportions of CNT. The reference sample was made of only a pultruded GFRP profile. In the study, the reference sample and four samples with CNT were tested under compression. The largest buckling load difference between the reference sample and the sample with CNT was 37.7%. The difference between the analytical calculation results and experimental results was obtained with an approximation of 0.49%–4.92%. Finally, the buckling, debonding, interlaminar cracks, and fiber breakage were observed in the samples.

## 1 Introduction

Composite materials are now widely used because they may provide improved qualities compared to conventional materials, which are becoming more important as lightweight designs become more important in terms of the energy efficiency and ergonomics [[1], [2], [3], [4]]. Matrix and reinforcing elements are the two main components of composite materials [[5], [6], [7], [8]]. It is often a matrix made up of epoxy-type polymer resins, reinforcing fibers, and nanoparticles. Strength comes from reinforcement, which is often brittle and rigid. The reinforcements are held together by the matrix, which also offers the strength and ductility. Composite materials can be produced according to the desired conductivity properties as well as high stiffness/density ratios and high physical properties [9]. Thus, stronger and lighter structures are obtained. The most striking example of this can be seen in the aviation industry.

A structural sandwich consists of a thick, somewhat weak core sandwiched between two hard, thin surfaces [10,11]. The compression of the core between the lower and upper surfaces and the integrated connection of the two surfaces result in a structure with excellent bending stiffness and low weight [12]. Two thin surface composite materials are sandwiched together to create sandwich composite structures, which offer excellent stiffness and low weight. Due to their higher energy absorption properties, sandwich composite constructions are utilized [13,14]. Structurally, the core serves a variety of functions. First, it provides continuous support for the faces. The tensile and compressive loads are first carried by the bottom and upper surfaces that make up the sandwich structure. The sandwich structure's thickness and the transverse shear stresses are carried by the honeycomb that makes up the core structure [15]. The core structure also significantly increases the moment of inertia, bending stiffness in all directions, and impact the energy absorption. Furthermore, it needs to be thick enough to offer high shear stiffness and strong enough to endure transverse shear loads. Compared to the faces, the core experiences substantially less compressive and tensile loads. One of the positive effects of composite materials is the large number of matrix resins and reinforcing elements (fibers) available to meet the design requirements. Commonly used fiber materials include glass fiber, aramid fiber, and carbon fiber [[16], [17], [18], [19]].

Large interlayer regressions take place, which cause delamination at the interface section between the layers if the mechanical characteristics of the laminated composite remain unchanged. To avoid this, functionally graded materials (FGMs), whose material characteristics are continually changing, or graded materials with the same or similar mechanical properties are employed as coatings [20,21]. This is done by gradually changing the volume ratios of the component material along the direction of thickness. As a result, the interface issues with composite materials are resolved, and the stress distribution is smoothed down. FGMs are composite structures that do not exhibit homogenous qualities; instead, they demonstrate progressive positional variations in the material properties as a result of the function that determines grading [22,23]. FGMs are a unique class of composite materials whose component volume ratios are constantly changing from one side to the other [24].

Utilizing nanomaterials as reinforcement offers a potential avenue for enhancing the characteristics of composite materials by converting the reinforcement phases into nanoscale fillers [[25], [26], [27], [28], [29]]. Carbon nanotube (CNT) is one of the nanomaterials used in many applications and is still under development [30,31]. Given the expensive cost of CNTs, the best possible application of a given amount of reinforcement in polymer samples is crucial. Researchers from both domestic and international institutions are interested in functionally graded CNT-reinforced composites (FG-CNTRCs), a novel class of materials made possible by the linear distribution of CNTs in a matrix [32]. In traditional composites, the material design with mechanical qualities in mind is altered to achieve the desired mechanical properties by reversing the fiber orientations [33,34]. When looking at fiber-reinforced polymer (FRP) composites as a whole, it becomes crucial to reinforce the interlayer area that is referred to as the weak link [35,36]. This issue is solved by the fibers' random dispersion rather than layering structure.

The mechanical characteristics of nanocomposites were then investigated employing FG-CNTRC beams, plates, and shell structures [[37], [38], [39], [40], [41], [42]]. Srivastava et al. [43] studied the buckling behavior of nanocomposite plates with randomly arranged nanotubes under various stress conditions. Song et al. [44] explored biaxially compressed functionally graded graphene nanoplatelets-reinforced composite plates. The analysis of FG-CNTRC shells, plates, and beams has received significant attention from Liew et al. [17]. Jalali et al. [45] evaluated the buckling of FG-CNTRC with circular sandwich plates. Plates sitting on a Pasternak elastic base were subjected to static and dynamic examinations. In order to determine the mechanical behavior of textile composites reinforced with CNTs and their impact on elastic characteristics, Gojny et al. [46] assessed the effect on the mechanical characteristics of epoxy-based nanocomposites, using single, double, and multi walled CNTs. The highest increases were obtained with amino-functionalized double-walled carbon nanotubes with 0.5 wt percent filler: 10% in terms of the tensile strength, 15% in terms of the stiffness, and 43% in terms of the fracture toughness. The free vibration analysis of FG-CNTRC beams, as reported by Madenci [47], is done within the context of variational formulations. The effective material characteristics of SWCNT reinforced nanobeams are estimated utilizing the rule of mixing. These studies collectively contribute to understanding the mechanical behavior of FG-CNTRCs under various loading conditions, emphasizing the importance of considering different deformation effects on the analysis of such structures. Future research could focus on refining micromechanical models, exploring additional loading scenarios, and investigating the influence of environmental conditions on the mechanical properties of FG-CNTRCs.

Numerous micromechanical material models have been utilized to predict the effective material properties of FG-CNTRCs. These models encompass the Halpin-Tsai model, Mori-Tanaka model, Rule of mixture, and the Eshelby-Mori-Tanaka approach. Shen and Zhu [48] evaluated the pre- and post-buckling behaviors of simply supported FG-CNTRC plates under uniaxial compression at different temperatures. They subsequently conducted post-buckling analysis on perfect and imperfect FG-CNTRC cylindrical shells subjected to combined axial and radial loads, as well as torsion under thermal loading, employing a multi-scale method and micromechanical model.

In the analysis of such structures, it is essential to account for shear deformation effects. Therefore, the use of the first-order shear deformation theory (e.g., Timoshenko theory) and higher-order shear deformation theories is recommended [[49], [50], [51], [52], [53], [54]]. Mirzaei et al. [55] utilized Timoshenko beam theory and the Ritz technique with polynomial basis functions to explore the nonlinear free vibration of CNTRC sandwiches. Zhu et al. [56] assessed nanocomposite plates reinforced with straight CNTs under static and free vibration conditions using the first-order shear deformation theory. Additionally, Mehar et al. [57] examined the free vibration behavior of FG-CNTRCs in a thermally active environment.

In this study, the buckling behavior of composite sandwich beams comprising a pultruded glass fiber-reinforced polymer (GFRP) core material and CNT reinforced laminated carbon fiber composite sheaths is investigated. The experimental and analytical evaluations of the load–deflection behavior and damage analysis of these sandwich beams under static buckling conditions are presented. By utilizing parameters obtained from coupon tests on the skin and core materials, the static behavior of the FG-CNTRC sandwich beams is theoretically predicted and compared to experimental results. The investigation draws on the use of CNTs to improve the thermo-mechanical properties of FRP composites, aiming to increase the critical buckling temperature of sandwich structures. The study involves testing a reference sample composed solely of a pultruded GFRP profile alongside four samples incorporating varying proportions of CNT under compressive loading. The results reveal a significant difference of 37.7% in the highest buckling load between the reference sample and the CNT-reinforced samples. Moreover, the comparison between the analytical calculations and experimental outcomes shows a close approximation ranging from 0.49% to 4.92%. The observed failure modes in the samples include the buckling, debonding, interlaminar cracks, and fiber breakage, highlighting the complex failure mechanisms present in these composite sandwich beams. This research contributes to the understanding of the buckling response of FG-CNTRC sandwich beams and emphasizes the importance of considering CNT reinforcement in enhancing the mechanical performance of composite structures under compressive loading conditions. Further exploration in this area could focus on optimizing the distribution of CNTs within the composite beams to improve their structural integrity and performance under various loading scenarios.

## 2 Materials

### 2.1 Specimen design

**Fig. 1:**
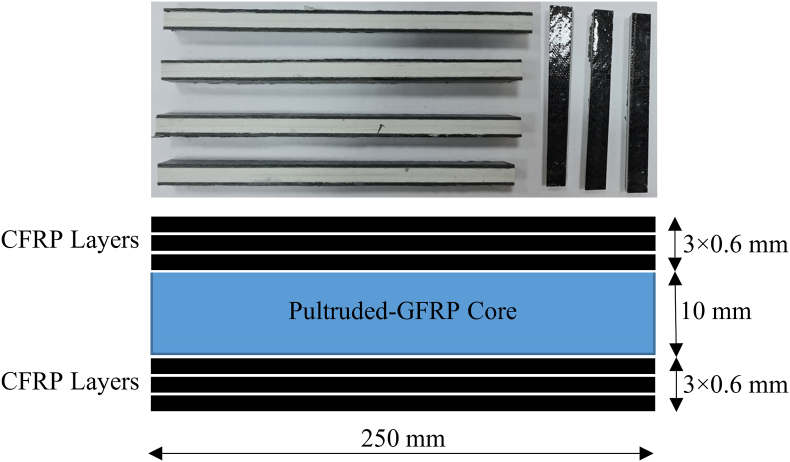
Test specimens and geometry.

Sandwich beams were made of composite coatings with carbon fiber layer reinforced with CNT on GFRP core material produced by the pultrusion method. Pultruded GFRP profiles have recently been utilized in different engineering applications [[58], [59], [60], [61], [62]]. Four FGM composites having different configurations were tested under axial loads. For all the specimens, pultruded lamina was used as a core element. Three carbon fiber-reinforced polymer (CFRP) layers were bonded to below of this core element and three layers were bonded upper side (Fig. 1).

The specimen designs are presented in Table 1. For the first specimen (FGM1), no CNT was included in the CFRP layers. For the second specimen (FGM2), the CNT ratios of 0.3%, 0.4%, and 0.5% from upper to lower CFRP were utilized. Symmetrical distribution was selected for the bottom part. For the third specimen (FGM3), the CNT ratios of 0.5%, 0.4%, and 0.3% from upper to lower CFRP were used, and symmetrical distribution was selected for the bottom part. For the fourth specimen (FGM4), the CNT ratios of 0.3%, 0.4%, and 0.5% from upper to lower CFRP were employed, and anti-symmetrical distribution was selected for the bottom part. The thickness of each layer was 0.6 mm. The pultruded thickness using on a core region was 10 mm. The cross-sectional area of each sample was 25 mm × 13.6 mm.

**Table 1:** Design of specimens.

0% CNT	0.3% CNT	0.5% CNT	0.3% CNT
0% CNT	0.4% CNT	0.4% CNT	0.4% CNT
0% CNT	0.5% CNT	0.3% CNT	0.5% CNT
**Core**	**Core**	**Core**	**Core**
0% CNT	0.5% CNT	0.3% CNT	0.3% CNT
0% CNT	0.4% CNT	0.4% CNT	0.4% CNT
0% CNT	0.3% CNT	0.5% CNT	0.5% CNT
**FGM1**	**FGM2**	**FGM3**	**FGM4**

#### 2.1.1 CNT reinforced CFRP sheets

The epoxy resin utilized in this study has a viscosity of 600–900 mPas, is two-phase, and comprises a combination of 10%–20% aliphatic diglycidyl ether and 80%–90% diglycidyl ether bisphenol A. In this research work, multi-walled carbon nanotubes (MWCNTs) were chosen because they are more affordable and evenly dispersible in epoxy resins. Tenax-E HTA 40 3k yarn was used to create a 200 gr carbon fiber cloth in a plain weave. For applications where lightweight, strength, and carbon are crucial, CFRP is perfect. In Table 2, mechanical characteristics are listed. Three layers of CFRP were employed.

**Table 2:** Material properties of CNT, CFRP, and epoxy.

Material	Tensile strength (MPa)	Modulus of elasticity (kPa)	Elongation at break (%)	Density (g/cm^3^)
Epoxy	70–80	3–3.3	5–6.5	1.18–1.20
CNT	10–60	1	1.3–2	10
CFRP	3950	238	1.7	1.76

**Fig. 2:**
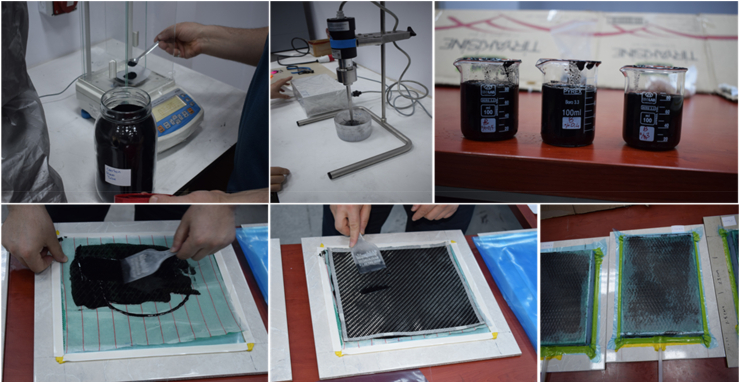
Manufacture of FG-CNTRC sheets.

By employing the vacuum infusing technique to symmetrically layer three layers of CFRP on the mold and inject resin into it, epoxy matrix carbon fiber reinforced composite plates were created. The composite plates were created and allowed to cure for 24 h at room temperature. For nanoparticles made of three layers of CFRP, the same technique was applied. At this point, 0.3, 0.4, and 0.5% by weight of MWCNTs were introduced into the epoxy matrix. In the initial phase, epoxy resin and CNT powder were stirred together using a mechanical stirrer for 15 min. After utilizing a mechanical stirrer in the first phase, an ultrasonic homogeneity equipment was employed. The infusion of nano-reinforced resin into CFRP was accomplished through a vacuum infusion process (Fig. 2.).

### 2.2 Pultruded GFRP core

Due to additional production processes like fiber wrapping and resin transfer molding, the resistance of composite material building parts to incoming loads is constrained. This situation has sparked the search for inventive production techniques. The pultrusion technique was developed to make profile pieces with low production costs, straightforward mass production, and high strength values. In the pultrusion process, continuous fibers are often used as reinforcing material and are combined to be utilized in woven or broken felts and roving. Most often, polyester is employed as a matrix material. In this experiment, the sandwich beams' cores were made of pultruded GFRP composite. The longitudinal and transverse young modules were reported as 23 GPa and 7 GPa, respectively. On the other hand, the longitudinal and transverse tensile strengths were reported as 240 MPa and 50 MPa, respectively.

## 3 Theoretical formulation

The buckling behavior of functionally graded sandwich beams is a critical aspect in the design and analysis of advanced composite structures. In recent studies, researchers have used a higher-order shear deformation theory to evaluate the buckling responses of these beams. This theory allows for a more accurate representation of the structural behavior under various loading conditions, considering the complexities of shear deformation effects. Understanding the buckling characteristics of functionally graded sandwich beams is essential for optimizing their performance and ensuring structural integrity.

The buckling responses of functionally graded sandwich beams have been analyzed using a higher-order shear deformation theory. The displacement field for this investigation is provided as follows [63]:

(1) $$\begin{array}{l} u\left(x,z,t\right)=u_{0}\left(x,t\right)-z\frac{\partial w_{0}}{\partial x}+f\left(z\right)\varphi_{x}\left(x,t\right)\\ w\left(x,z,t\right)=w_{0}\left(x,t\right) \end{array}$$

where “u” and “w” represent the displacement components through the “x” and “z” coordinate directions, respectively, whereas “$u_{0},w_{0}$” and “$\varphi_{x}$” are the unknown variables of middle surface of the FG-CNTRC sandwich beam. Hence, f(z) indicates a shape function in terms of thickness coordinate “z” that governs the distribution of the transverse shear strains and stresses in the thickness direction. In the context of high-order shear deformation theory, the shape function plays a crucial role in capturing the intricate distribution of the transverse shear strains and stresses along the thickness direction of the material. By incorporating a high-order polynomial shape function, the theory can provide a more accurate representation of the structural response under various loading conditions, enhancing the predictive capabilities of the analysis. This approach allows for a detailed examination of the complex interplay between the deformation and stress distribution within the material, offering valuable insights into its mechanical behavior. In this study, we adopted the polynomial shape function by setting:

(2) $$f\left(z\right)=\frac{7}{4}z-\frac{7}{3}\frac{z^{3}}{h^{2}}$$

The kinematic relations, which describe the motion and deformation of a material, are fundamental in understanding the behavior of structures under various loading conditions. These relations can be derived through mathematical formulations that link the displacement, velocity, and acceleration fields within the material. The kinematic relations can be obtained as below [64]:

(3) $$\begin{array}{l} \varepsilon_{x}=\varepsilon_{x}^{0}+z\varepsilon_{x}^{1}+f\left(z\right)\varepsilon_{x}^{2}\\ \gamma_{xz}=g\left(z\right)\gamma_{xz}^{0} \end{array}$$

where

(4) $$\begin{array}{l} \varepsilon_{x}^{0}=\frac{\partial u}{\partial x},\;\varepsilon_{x}^{1}=-\frac{\partial^{2}w_{0}}{\partial x^{2}},\;\varepsilon_{x}^{2}=\frac{\partial^{2}\varphi_{x}}{\partial x^{2}}\\ \gamma_{xz}^{0}=\frac{\partial \varphi_{x}}{\partial x} \end{array}$$

The formulation of equations governing the behavior of a beam under varying load effects involves a comprehensive analysis of the axial and transverse stress components. The equations governing the behavior of the beam under load effect variations, with a focus on the axial and transverse stress components, can be formulated as [65]:

(5) $$\begin{array}{l} \sigma_{x}=Q_{11}\left(z\right)\varepsilon_{x}\\ \tau_{xz}=Q_{55}\left(z\right)\gamma_{xz} \end{array}$$

where *Q*_11_ and *Q*_55_ are evaluated in terms of the material constants as:

(6) $$\begin{array}{l} Q_{11}=E_{11}\\ Q_{55}=G_{13} \end{array}$$

The potential energy principle using virtual displacements is harnessed to derive the governing equilibrium equations in a variational format. This approach allows for a comprehensive analysis of the beam's behavior under varying load effects, focusing on the axial and transverse stress components. By formulating these equations, a detailed understanding of the structural response to different loading conditions can be achieved, providing insights into the beam's mechanical performance and deformation characteristics. Leveraging the principles of the total potential energy and virtual displacements offers a robust framework for predicting the beam's equilibrium behavior and stress distribution, improving the accuracy of the structural analysis and design methodologies. In this study, the total potential energy principle employing virtual displacements was utilized to derive the equilibrium equations in a variational format [66].

(7) ∫−h2h2∫A(σxδεx+τxzδγxz)dAdz−∫Aqδw0+∫ANx0∂w0∂x∂δw0∂xdA=0

in which “*A*” is the top surface of the plate, “*q*” and “$N_{x}^{0}$” are the transverse and in-plane distributed loads, respectively. By substituting Eq. (3) into Eq. (5), the total potential energy principle can be rewritten in the following form [66,67]:

(8) $$\int_{A}\left\{N_{x}\delta \varepsilon_{x}^{0}+M_{x}^{b}\delta \varepsilon_{x}^{1}+M_{x}^{s}\delta \varepsilon_{x}^{2}+S_{xz}^{s}\delta \gamma_{xz}^{0}-q\delta w_{0}+N_{x}^{0}\frac{\partial w_{0}}{\partial x}\frac{\partial \delta w_{0}}{\partial x}\right\}dA=0$$

where “$N_{x}$”, “$M_{x}^{b}$”, “$M_{x}^{s}$”, and “$S_{xz}^{s}$” are the stress resultants which can be expressed by the following relations [67,68]:

(9) Nx=∫−h2h2σxdz;Mxb=∫−h2h2σxzdz;Mxs=∫−h2h2σxfzdz;Sxzs=∫−h2h2τxzgzdz

By substituting the constitutive relations of Eq. (5) into Eq. (9), the stress resultants can be found in terms of generalized strains as follows [67,69]:

(10) $$\begin{array}{l} N_{x}=A_{11}\varepsilon_{x}^{0}+B_{11}\varepsilon_{x}^{1}+E_{11}\varepsilon_{x}^{2}\\ M_{x}^{b}=B_{11}\varepsilon_{x}^{0}+D_{11}\varepsilon_{x}^{1}+F_{11}\varepsilon_{x}^{2}\\ M_{x}^{s}=E_{11}\varepsilon_{x}^{0}+F_{11}\varepsilon_{x}^{1}+H_{11}\varepsilon_{x}^{2}\\ S_{xz}^{s}=A_{55}^{s}\gamma_{xz}^{0} \end{array}$$

where “$A_{11}$”, “$B_{11}$”, “$D_{11}$”, “$E_{11}$”, “$F_{11}$”, “$H_{11}$”, and “$A_{55}^{s}$” are the plate stiffness defined by [64,67].

(11) (A11,B11,D11,E11,F11,H11)=∫−h2h2Qij(1,z,z2,f(z),zf(z),f2(z))dzA55s=∫−h2h2Qijg2(z)dz

By substituting Eqs. (3) and (5) into Eq. (7) and integrating by parts with respect to “x” and setting the coefficients of “$\delta u_{0}$”, “$\delta w_{0}$”, and “δφ” to zero, separately, the governing equilibrium equations of the stability in terms of the stress resultants are found below:

(12) $$\begin{array}{l} \delta u_{0}:\frac{\partial N_{x}}{\partial x}=0\\ \delta w_{0}:\frac{\partial^{2}M_{x}^{b}}{\partial x^{2}}+N_{x}^{0}\frac{\partial^{2}w_{0}}{\partial x^{2}}+q=0\\ \delta \varphi :\frac{\partial^{2}M_{x}^{s}}{\partial x^{2}}-\frac{\partial S_{xz}^{s}}{\partial x}=0 \end{array}$$

The Navier's solution procedure is employed to analyze the buckling behavior of a simply supported FG-CNTRC sandwich beam under applied forces. This classical analytical method allows for the determination of critical buckling loads and modes of the sandwich beam structure. By utilizing the Navier's solution procedure, the study aims to provide insights into the stability characteristics of FG-CNTRC sandwich beams, offering valuable information for optimizing their design and structural performance. The application of this established solution technique enhances understanding of the buckling response of FG-CNTRC sandwich beams, contributing to the advancement of the composite material analysis and design methodologies. The Navier's solution procedure is used for the buckling analysis of simply supported FG-CNTRC sandwich beam subjected to forces ($N_{x}^{0}$). However, in the case of static buckling problem, all other forces acting on the beam are assumed to be zero (q = 0). Based on the Navier's procedure, the solution of the unknown variables satisfying the above boundary conditions yielded can be represented in the double trigonometric form as follows [70,71]:

(13) $$\left\{\begin{array}{l} u_{0}\\ w_{0}\\ \varphi \end{array}\right\}=\sum_{m=1}^{\infty }\left\{\begin{array}{l} U_{m}\;\cos\left(\lambda x\right)\\ W_{m}\;\sin\left(\lambda x\right)\\ \Phi_{m}\;\sin\left(\lambda x\right) \end{array}\right\}$$

## 4 Experimental program

**Fig. 3:**
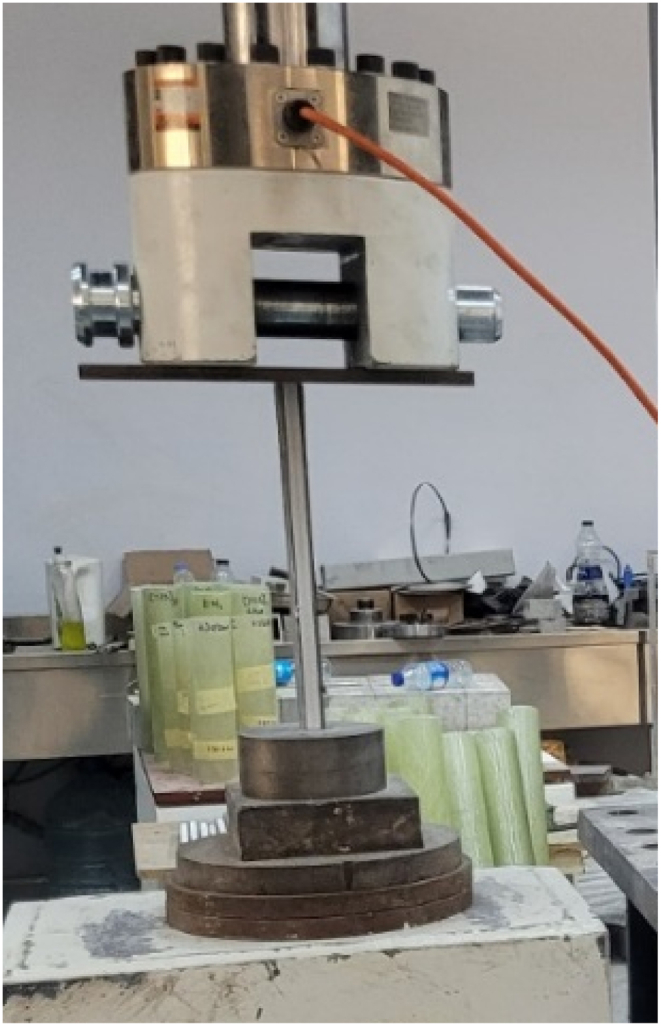
Test setup.

Buckling tests were performed under compressive loading. The length of the specimens was 250 mm. The ends of the specimens were not explicitly restrained. The test setup is displayed in Fig. 3. The specimens were loaded at the speed of 1 mm/min. Loading and vertical displacements were recorded during the experiments. Three repetitions were utilized in order to get accurate results. Since no restraints were applied, the buckling length of the specimens was 250 mm.

## 5 Results and discussion

When designing a rod under pressure, two important conditions are encountered. The first one is the stress problem, where the maximum stresses are expected to be lower than the safety stress. The second one is the stability problem, where the stability of the equilibrium is expected. If the equilibrium state is not stable, the support system will collapse. During the stress problem analysis, the stability of the known equilibrium state is investigated. In pressure-loaded elements, buckling is one of the most critical problems.

**Fig. 4:**
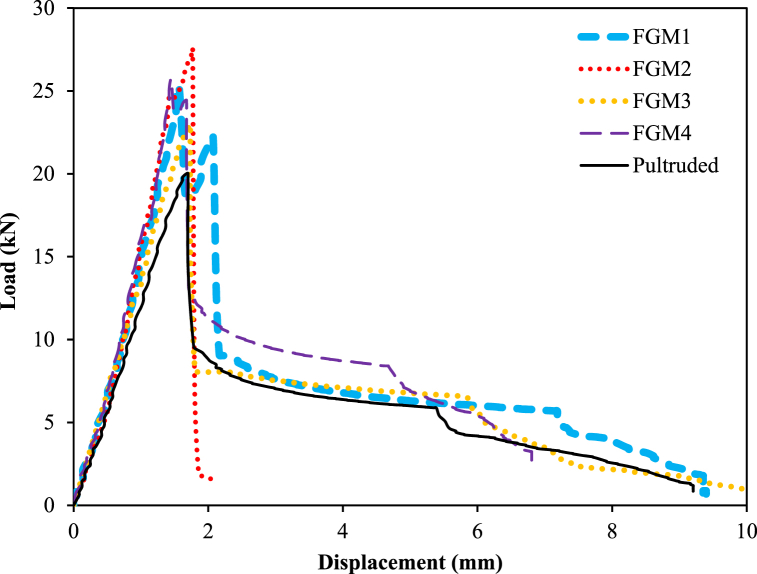
Load–displacement comparison of samples.

In this experimental study, each sample had a width of 13.6 mm and a length of 25 mm, with a total height considered as 250 mm. Each sample was placed in a simply supported manner in the loading device. Therefore, the rod length and buckling length were assumed to be equal in the calculations. In other words, the results demonstrated that in the FG-CNTRC samples, the buckling mode was sensitive to the placement distribution of CNTs. The average load–displacement relationship for a total of five samples (FGM1, FGM2, FGM3, FGM4, and pultruded) subjected to buckling is depicted in Fig. 4. The average buckling load capacities of the samples were 25.22 kN for FGM1, 27.57 kN for FGM2, 22.98 kN for FGM3, 25.66 kN for FGM4, and 20.01 kN for the pultruded sample.

Upon examining Fig. 4, it can be observed that the maximum load-carrying capacity was highest in FGM2 and lowest in the pultruded sample. In other words, compared to the pultruded sample, FGM1, FGM2, FGM3, and FGM4 carried 26.00%, 37.70%, 14.80%, and 28.20% more load, respectively. When the displacements corresponding to the maximum load values are compared to the pultruded sample, it is revealed that FGM4 experienced a 14.7% maximum decrease, while FGM2 exhibited a 4.1% maximum increase. Finally, when comparing the stiffness values corresponding to the maximum load values, the following relationship is evident: pultruded < FGM3 < FGM2 < FGM1 < FGM4.

All these parametric values depend noticeably on the CNT content, interlayer distribution of CNT, and adhesion success of CFRP to the pultruded element. Table 3 provides the maximum load value and the corresponding displacement for each sample. Additionally, the displacement and stiffness values corresponding to the maximum loads are presented in the table. Fig. 4 illustrates the load–displacement comparisons.

**Table 3:** Maximum load-carrying capacities and displacements at maximum loads.

Sample name	Maximum load (kN)	Rate of increase (%)	Displacement at maximum load (mm)	Rate of increase (%)	Stiffness at maximum load (kN/mm)
Pultruded	20.01	–	1.70	–	11.77
FGM1	25.22	26.00	1.56	−8.20	16.66
FGM2	27.57	37.70	1.77	4.10	15.57
FGM3	22.98	14.80	1.73	1.70	13.28
FGM4	25.66	28.20	1.45	−14.7	17.69

**Fig. 5:**
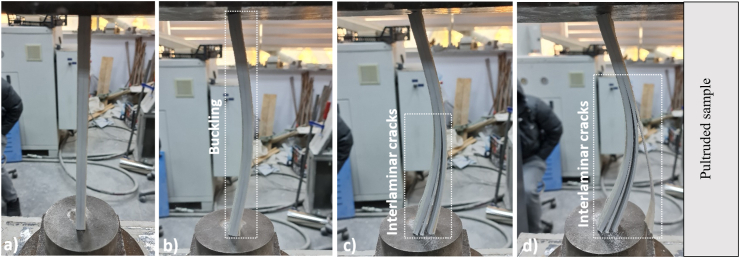
Damage analysis of pultruded sample.

**Fig. 6:**
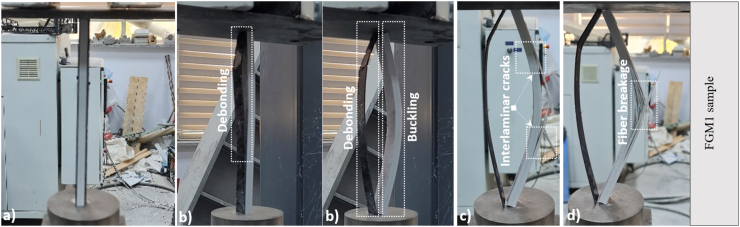
Damage analysis of FGM1 sample.

Each specimen placed simply supported had two moments of inertia (I_min_ = 5240.53 mm^4^ and I_max_ = 17708.33 mm^4^). As it is known, buckling occurs in the direction of the axis where the moment of inertia is small. When the experimental samples were examined, all the samples were buckled in the weak axis direction. The slenderness ratio (λ) of the samples was determined as 64.67 as a result of the ratio of the buckling length of the bar (L = 250 mm) to the radius of inertia (i) of the bar. While performing the damage analysis of the samples, initial appearances (Fig. 5a), buckling load moment or CFRP state separated from the pultruded element (Fig. 5b), pultruded core fracture (interlaminar cracks) (Fig. 5c), and advanced damage state after buckling and final state (Fig. 5d) were witnessed. When the pultruded specimen, displayed in Fig. 5, was assessed, buckling occurred when the maximum load was reached. Afterward, interlaminar cracks started from the bottom point and formed at the middle height of the sample in the longitudinal direction, and the test was terminated. When the FGM1 sample given in Fig. 6 is evaluated, the first damage was observed as a debonding between CFRP and the pultruded core in the region from the top to the middle of the sample. At the same time, the CFRP sheet on the other side of the sample was completely separated. Then, buckling occurred as soon as the debonding damage was seen at half-height of the sample progressed rapidly along the sample height. In FGM1, the vertical displacement increased after buckling, and failure occurred by observing the interlaminar cracks and fiber breakage at the midpoint of the sample (Fig. 6).

**Fig. 7:**
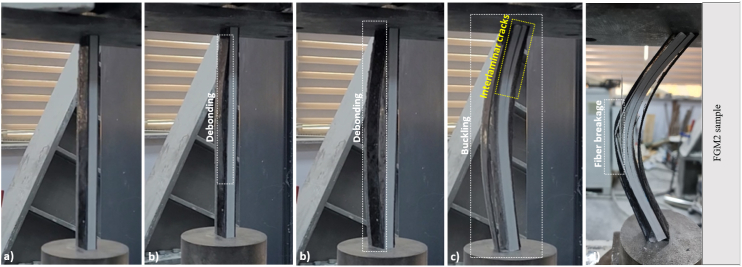
Damage analysis of FGM2 sample.

In FGM2, the debonding damage was first observed in the CFRP sheet on the left side of the pultruded core element. Later, when FGM2 reached the buckling load, interlaminar cracks abruptly formed in the upper part. With the increase in the vertical displacement, the fiber breakage occurred in the buckling direction and the experiment was terminated (Fig. 7).

**Fig. 8:**
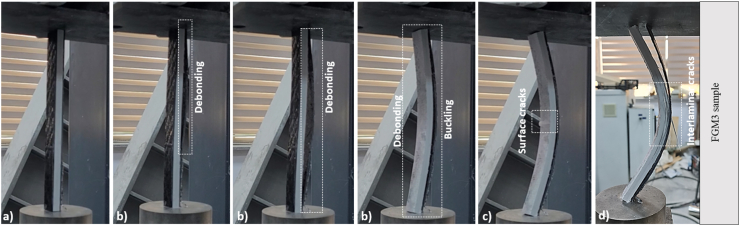
Damage analysis of FGM3 sample.

The damage in the FGM3 sample primarily started with the observation of debonding in the CFRP sheet on the right surface of the pultruded core element. However, all of a sudden, the CFRP sheet on the left surface was damaged by debonding and separated from the pultruded core element and buckling occurred. With the increase in the vertical displacement, damage occurred on the left surface of the pultruded core element in the middle of the height. As the displacement increased, the interlaminar cracks were formed on the right surface of the pultruded core element and collapse occurred (Fig. 8).

**Fig. 9:**
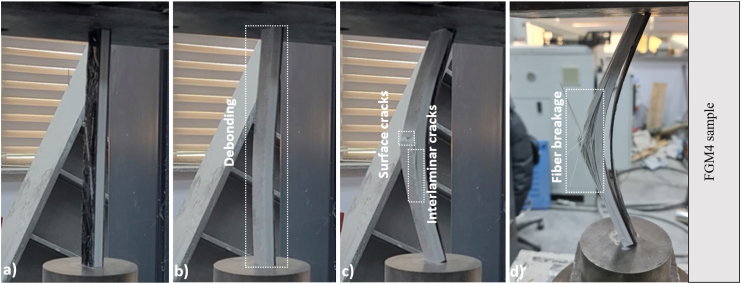
Damage analysis of FGM4 sample.

Finally, when the FGM4 sample in Fig. 9 was evaluated, the first damage occurred due to the debonding damage of the CFRP sheet on the left surface of the pultruded element. The final damage of the sample, whose load-carrying capacity started to decrease with buckling, was realized with the fiber breakage.

The comparison of the samples for which experimental studies and analytical calculations were made is given in Table 4. It is understood that the experimental and analytical results are in acceptable agreement.

**Table 4:** Experimental and analytical comparisons of samples.

Load (N)	FGM1	FGM2	FGM3	FGM4	Pultruded
Experimental	25.22 ± 0.38	27.58 ± 1.16	22.99 ± 1.41	25.67 ± 0.81	20.01 ± 0.55
Analytical	26.24	29.01	24.63	26.42	20.11
Difference (%)	3.88	4.92	6.6	2.8	0.49

By comparing the buckling characterizations of FG-CNTRCs in the experiment, two distinct buckling modes corresponding to the local buckling and global buckling can be mentioned. However, when all the figures were examined, global buckling was observed clearly in all the samples.

## 6 Conclusions

Experimental and analytical results of the pultruded, FGM1, FGM2, FGM3, and FGM4 samples tested under compression were obtained. The following specific conclusions can be drawn by assessing the damage observed in the samples together with the results obtained:

• The CNT configuration of the CFRP sheet adhered to the right and left sides of the pultruded core had an effect on the load-carrying capacity and stiffness. While the first layer of CFRP sheet contacting the pultruded core had 0.5% CNT (FGM2), the highest buckling load (27.57 kN) was obtained. There was a relationship between the load-carrying capacities (buckling loads) of the samples as pultruded < FGM3<FGM1<FGM4<FGM2.
• When the displacements corresponding to the maximum load values were compared to those of the pultruded sample, a maximum decrease of 14.7% occurred in FGM4, while a maximum increase of 4.1% was seen in FGM2.
• The analytical calculations were compared to the experimental results with an approximation between 0.49% and 4.92%.
• The damages observed in the samples were generally in the form of the buckling, debonding, interlaminar crack, and fiber breakage.

As a result of the study, it was understood that the rate and location of CNT are important for FG-CNTRCs. In addition, it can be stated that experimental results can be supported by analytical results. For future studies, increasing the thickness of the core element, applying the loading only to the core element, and the core element being CFRP instead of GFRP can be investigated.

This study collectively enhance the comprehension of the mechanical behavior of FG-CNTRCs under diverse loading conditions and underscore the significance of considering shear deformation effects in the analysis of such structures. Future research in this domain could concentrate on improving these micromechanical models, exploring additional loading scenarios, and examining the impact of other factors like environmental conditions on the mechanical properties of FG-CNTRCs.

## Funding

The authors are thankful to the Deanship of Scientific Research at 10.13039/501100005911Najran University for providing financial support to this research work under the Research Groups Funding program grant code NU/RG/SERC/12/11 and 10.13039/501100012190Ministry of Science and Higher Education of the Russian Federation as part of the World-Class Research Center program, Advanced Digital Technologies (contract No. 075-15-2022-312 dated April 20, 2022).

## Data availability statement

The authors declare that the data supporting the findings of this study are available within the article.

## CRediT authorship contribution statement

**Emrah Madenci:** Writing – original draft, Validation, Methodology, Investigation, Conceptualization. **Yasin Onuralp Özkılıç:** Writing – review & editing, Writing – original draft, Validation, Methodology, Investigation, Conceptualization. **Alireza Bahrami:** Writing – review & editing, Writing – original draft, Validation, Resources, Methodology, Investigation, Formal analysis, Conceptualization. **Ceyhun Aksoylu:** Writing – review & editing, Writing – original draft, Validation, Formal analysis. **Muhammad Rizal Muhammad Asyraf:** Validation, Methodology, Investigation. **Ibrahim Y. Hakeem:** Validation, Formal analysis, Conceptualization. **Alexey N. Beskopylny:** Writing – review & editing, Validation, Investigation. **Sergey A. Stel'makh:** Writing – review & editing, Validation, Formal analysis. **Evgenii M. Shcherban:** Investigation, Formal analysis. **Sabry Fayed:** Validation, Methodology.

## Declaration of competing interest

The authors declare that they have no known competing financial interests or personal relationships that could have appeared to influence the work reported in this article.
